# Acenocoumarol-Induced Diffuse Alveolar Hemorrhage: A Case Report

**DOI:** 10.7759/cureus.37581

**Published:** 2023-04-14

**Authors:** Twinkle Pawar, Abhinav Ahuja, Tushar Sontakke, Anil Wanjari, Sourya Acharya, Sunil Kumar

**Affiliations:** 1 Department of Medicine, Jawaharlal Nehru Medical College, Datta Meghe Institute of Medical Sciences (Deemed to be University), Wardha, IND; 2 Department of Internal Medicine, Jawaharlal Nehru Medical College, Datta Meghe Institute of Medical Sciences (Deemed to be University), Wardha, IND

**Keywords:** corticosteroid, hemoptysis, pt-inr, diffuse alveolar hemorrhage, acenocoumarol

## Abstract

Diffuse alveolar hemorrhage (DAH) is bleeding into the alveolar spaces of the lung. DAH is often associated with systemic autoimmune diseases, coagulation disorders, drugs, inhaled toxins, or transplantation. This study describes a rare case of acenocoumarol-induced DAH, a pulmonary disorder, which has not been reported before. A 48-year-old male presented with a history of rheumatic heart disease with mitral stenosis with moderate mitral regurgitation status post mitral valve replacement. He was taking acenocoumarol but did not keep his prothrombin time-international normalized ratio (PT-INR) monitoring and came to the hospital with complaints of cough, hemoptysis, and breathlessness. Chest x-ray and high-resolution computed tomography (HRCT) thorax were done which revealed diffuse patchy opacities and pulmonary hemorrhage, respectively. After nine days of hospital stay with appropriate management with corticosteroids, antibiotics, and intravenous fluids, the patient was doing well.

## Introduction

Diffuse alveolar hemorrhage (DAH) is a threat to life, associated with pulmonary damage. Various medical anomalies contribute to DAH such as coughing up bloody sputum (hemoptysis), diffuse interstitial lung ailment along with malfunctioning of the respiratory system leading to its failure. The inception of DAH starts from deformity in small blood vessels, which are in close proximity to alveoli, and helps the process of diffusion and microcirculation. This includes capillaries in alveoli, small vessels known as arterioles, and venules. The causative list of DAH is long and contains varied factors. The narrowing of the blood vessels due to inflammation in them which can lead to necrosis is one of the reasons behind DAH. Other autoimmune disorders like rheumatoid arthritis, and Goodpasteur's disease. Although many other hemorrhages related to the pulmonary system should not be treated as DAH as both of them have different manifestations and fallout impacts. The conditions which are not initiated by the innate immune system response constitute drugs, intoxicants, bleeding disorders, and malignancy [1,2].

## Case presentation

A 48-year-old male presented with cough, hemoptysis around 200 mL, high-grade fever, and breathlessness for four days. He was a known case of rheumatic heart disease with mitral stenosis with moderate mitral regurgitation. Mitral valve replacement was done seven years back and he was on the tablet Acitrom (acenocoumarol) 3 mg once a day with regular compliance. The patient denied any history of diabetes mellitus, systemic hypertension, tuberculosis, or bronchial asthma. He was non-alcoholic and a non-smoker. Rapid antigen test and reverse transcriptase-polymerase chain reaction were done in view of COVID-19, which came out to be negative and the patient was admitted to the medicine ward.

On general examination, he was febrile with a temperature of 39,4°C, pulse rate at 116 bpm, and regular respiratory rate at 26 per minute, blood pressure of 120/80 mm Hg in the right arm supine position. His SpO_2_ level was 92% on room air. No pedal edema, jugular venous pressure not raised. No other signs of bleeding were seen. Electrocardiography was suggestive of sinus tachycardia. On auscultation, a metallic click was present without any murmur. On respiratory system examination, there were diffuse crackles over both lungs. Per abdominal examination was normal, soft, non-tender, no hepatosplenomegaly. The patient's laboratory findings were as described in Table 1.

**Table 1:** Laboratory investigations

Complete blood count	Hemoglobin – 10.7 g/dL
	Hematocrit – 42.5%
	Platelet count – 97,000
	Whole blood count – 3,500 cells/cumm
Liver function test	Total protein – 6.7 g/dL
	Albumin – 3.2 g/dL
	Aspartate aminotransferase – 346 U/L
	Alanine aminotransferase – 212 U/L
	Alkaline phosphatase -12 U/L
	Total bilirubin – 0.9 mg/dL
Kidney function test	Creatinine – 1.2 mg/dL
	Urea – 24 mg/dL
	Sodium – 141 mmol/L
	Potassium – 4 mmol/L
Coagulation profile	Prothrombin time – 83.1 s
	international normalised ratio – 7.45
	activated Partial Thromboplastin Time – 81.2 s
D-Dimer	218 ng/mL
Creatinine kinase- MB	32 IU/L
Troponin I	6.85 ng/mL
C-Reactive protein	15.41 mg/dL
Ferritin	1,000 mg/L

**Figure 1:**
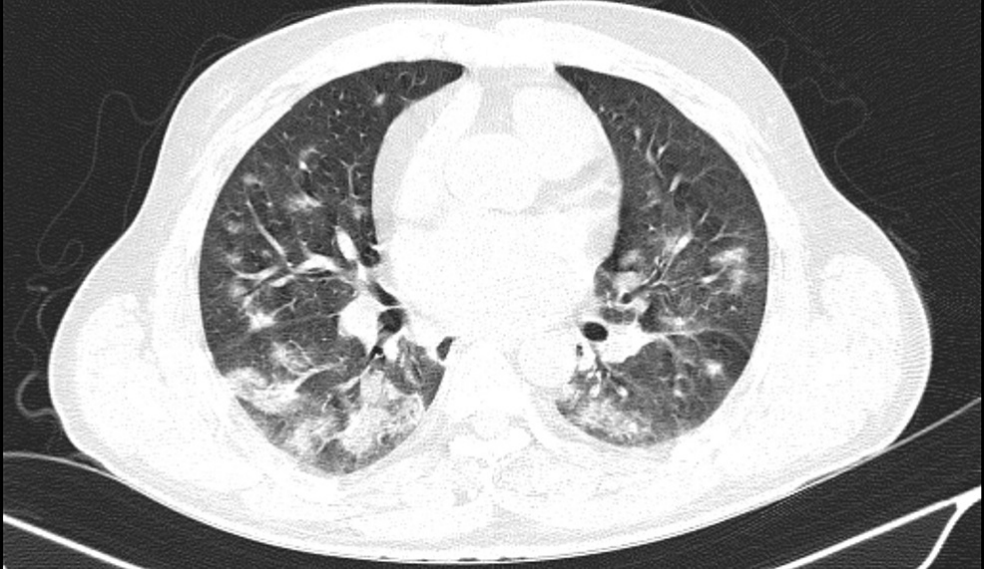
Round areas of ground glass opacities along with interlobular septal thickening predominantly in basal segments

Chest radiographs revealed that the lungs had bilateral patchy opacities. High-resolution computed tomography (HRCT) thorax was done which revealed multifocal rounded ground glass opacities scattered in bilateral lung parenchyma with subpleural sparing. There was also crazy paving with interlobular septal thickening suggestive of diffuse pulmonary hemorrhage (Figure 1). Human immunodeficiency virus, Hepatitis B, and Hepatitis C were negative. The patient was tested for scrub and leptospirosis, which was also negative.

**Figure 2:**
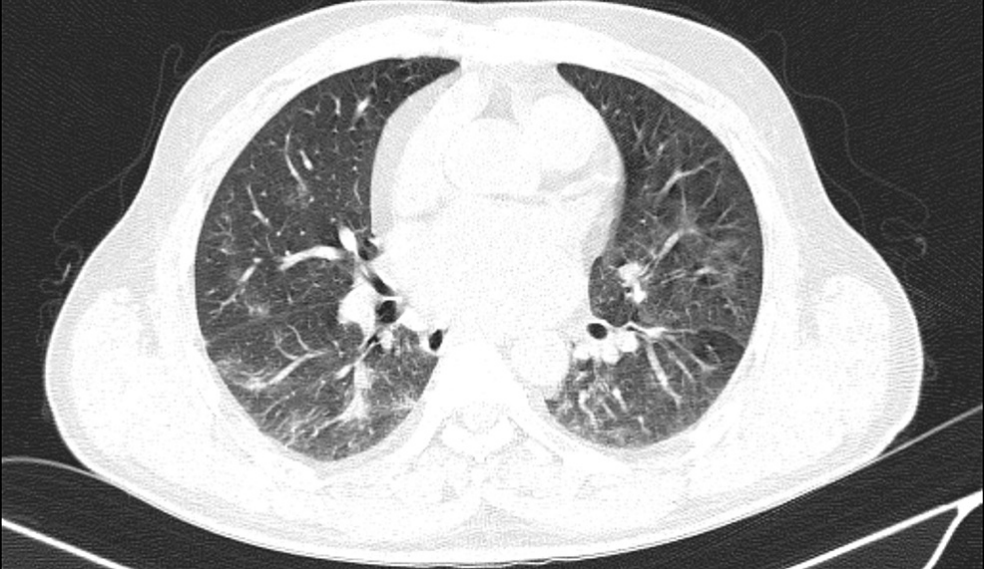
Same lesions showing significant resolution after one week

2D-Echo was done which was suggestive of prosthetic valve in situ, moderate aortic regurgitation, no aortic stenosis, moderate pulmonary artery hypertension with right ventricular systolic pressure of 40+ rap, no pericardial effusion or infective endocarditis. Acenocoumarol was withheld immediately, and the patient was started on injectable antibiotic in the form of ceftriaxone- sulbactam 1 g intravenously daily, diuretic as furosemide 40 mg, Injection vitamin K and inj methylprednisolone 250 mg. INR monitoring was done. After three days of withholding acenocoumarol, INR was found to be 2. A repeat x-ray and HRCT thorax were done in which pulmonary hemorrhage subsided (Figure 2). As we had kept the possibility of DAH due to acenocoumarol, the patient was switched to warfarin as further treatment. The patient was doing well on follow-up with warfarin after one month with INR maintained within the range of 2-2.5.

## Discussion

DAH is a sporadic situation taking place in medical exercise. It can be caused by various autoimmune disorders like rheumatoid arthritis, Goodpasteur's syndrome, systemic lupus erythematosus, antiphospholipid antibody syndrome, and Behçet's syndrome along with infections related to the pulmonary system, cardiac ailments such as mitral stenosis, clotting maladies, transplantation procedure of bone marrow or solid organ, poisonous contacts, adverse medication consequences of drugs like amiodarone, methotrexate, etc., and idiopathic pulmonary hemosiderosis. It has been noted that anticoagulants can cause DAH too [3]. Acenocoumarol acts as a vitamin K antagonist given in patients using metallic prosthetic heart valves to avert valve thrombosis and thromboembolic incidents with an aim of 3.0 (range 2.5 to 3.5) [4]. The key impediment of acenocoumarol therapy via the oral route is blood loss, particularly from important body parts for instance the lung or brain [5]. Alveolar hemorrhage is tough to detect and diagnose and has a high fatality rate if medical intervention is not taken in due time. Alveolar Hemorrhage may be mimicked as ground glass opacity on an HRCT scan. So, the patient must be worked up for the ground glass opacity which may be due to infective etiology like scrub typhus or leptospirosis or COVID-19 [6]. A few case reports have highlighted the complications of acitrom like coagulopathy in a case of atrial fibrillation with fast ventricular rate [7], and anticoagulant-related nephropathy in patients with aortic valve replacement [8]. Drug reaction with eosinophilia and systemic symptoms syndrome in a patient on acenocoumarol for the prevention of venous thromboembolism due to atrial fibrillation has been reported [9]. Case studies showed spontaneous epidural hematoma of the spine associated with acenocoumarol where all three patients were then treated with decompressive laminectomy [10]. The proposed hypothesis for this complication in acenocoumarol is coagulopathy due to deranged INR as happened in our patient. This deranged INR was due to liver injury, which was suggested by raised levels of transaminases and aspartate aminotransferase. These enzymes are sensitive indicators of drug-induced hepatocellular injury. Elevations in ALT and AST can occur from conditions other than liver injury, but ALT is relatively more specific because it is synthesized primarily by the liver. We suggest that the patients on anticoagulation medical intervention necessitate firms scrutinizing prothrombin time (PT) and international normalized ratio (INR) to shun severe blood loss difficulties associated with anticoagulation.

## Conclusions

DAH is often a life-threatening condition; a prompt diagnosis and early treatment are often required to decrease mortality. This case report highlights DAH after anticoagulation therapy as acenocoumarol; hence, these patients should require strict monitoring with PT/INR in order to avoid serious bleeding complications and prevent morbidity and mortality.
